# Endoscopic Technologies for Peripheral Pulmonary Lesions: From Diagnosis to Therapy

**DOI:** 10.3390/life13020254

**Published:** 2023-01-17

**Authors:** Alberto Fantin, Massimiliano Manera, Vincenzo Patruno, Giulia Sartori, Nadia Castaldo, Ernesto Crisafulli

**Affiliations:** 1 Department of Pulmonology, University Hospital of Udine (ASUFC), 33100 Udine, Italy; 2 Respiratory Medicine Unit, Department of Medicine, University of Verona and Verona University Hospital, 37134 Verona, Italy

**Keywords:** bronchoscopy, lung nodule, EBUS, TBNA, TBB, robotic bronchoscopy, ultrathin bronchoscopy, lung cancer

## Abstract

Peripheral pulmonary lesions (PPLs) are frequent incidental findings in subjects when performing chest radiographs or chest computed tomography (CT) scans. When a PPL is identified, it is necessary to proceed with a risk stratification based on the patient profile and the characteristics found on chest CT. In order to proceed with a diagnostic procedure, the first-line examination is often a bronchoscopy with tissue sampling. Many guidance technologies have recently been developed to facilitate PPLs sampling. Through bronchoscopy, it is currently possible to ascertain the PPL’s benign or malignant nature, delaying the therapy’s second phase with radical, supportive, or palliative intent. In this review, we describe all the new tools available: from the innovation of bronchoscopic instrumentation (e.g., ultrathin bronchoscopy and robotic bronchoscopy) to the advances in navigation technology (e.g., radial-probe endobronchial ultrasound, virtual navigation, electromagnetic navigation, shape-sensing navigation, cone-beam computed tomography). In addition, we summarize all the PPLs ablation techniques currently under experimentation. Interventional pulmonology may be a discipline aiming at adopting increasingly innovative and disruptive technologies.

## 1. Introduction

### 1.1. The Definition of PPLs

Peripheral pulmonary lesions (PPLs) are focal radiographic opacities undetectable by flexible bronchoscopy direct inspection [1,2]. Detection and diagnosis of PPLs represent a serious challenge for radiologists, thoracic surgeons, and respiratory physicians. The radiologic detection of PPLs has significantly increased during the last decades owing to the implementation of lung cancer screening programs [3]. Nowadays, PPLs are frequently incidental findings. They are reported in 0.2% of chest radiographs [4,5] and 13% of routinely performed chest computed tomography (CT) scans [6].

### 1.2. Evaluation and Risk Assessment of PPLs

PPLs may correspond to benign and malignant entities localized at the periphery of the lung. According to the National Lung Screening Trial findings, the early diagnosis of PPLs is associated with a 20% reduction in lung-cancer-specific mortality [7]. Thanks to this and other studies [8,9], guidelines have been proposed that recommend an annual low-dose chest CT scan screening for high-risk individuals (ages 55 to 77 years with ≥30 pack-year history of smoking, who are current smokers or have quit within the past 15 years) [10].

Histological diagnosis is of paramount importance to early detect malignant lesions. The decision whether to proceed to the histological sampling of the PPL depends on the radiographic appearance of the lesion and the presence of various patient’s related risk factors. Hence, when a PPL is identified, it is necessary to proceed with a risk stratification based on the patient profile and the characteristics found on chest CT. Patients with PPL are often asymptomatic, and when symptoms are present, they may reflect the etiologic condition underlying the development of the PPL [11]. Risk factors such as smoking, history of lung diseases, or malignancy need to be considered [12,13,14,15,16,17,18]. It is critical to recognize nodules with a high likelihood of being malignant and require further examination, while avoiding unneeded investigations for nodules with a lesser likelihood of cancer.

Primary lung cancer comprises an extensive range of histological types [19,20]. The primary historical division considered the distinction between small-cell lung cancer (SCLC) and nonsmall-cell lung cancer (NSCLC), the latter of which makes up about 85% of lung histotypes [21].

Among NSCLC, we should further categorize a few histotypes: squamous cell carcinoma (derived from squamous epithelial cells and mostly centrally located) [22,23], adenocarcinoma (which has a tendency to be peripheral and derives from bronchial mucosal tissue or alveolar surface epithelium), and large-cell carcinoma [24]. Among the ones cited, adenocarcinoma is the most common histotype of malignant PPLs [24]. Regarding the prognosis of malignant PPLs, data on this appear to be mixed, and undoubtedly histologic differentiation seems to play a role [22,23,24,25,26,27,28,29,30,31,32,33]. In many studies, no comparison is made between malignant lesion’s peripheral and central locations. As of today, it appears clear that histological subsets of malignant PPLs tend to have a faster growth rate than central ones [25,26,27,34], although there appears to be no difference in the chemotherapy failure rate [27,33,35]. Moreover, centrally located malignant lesions are frequently inoperable or unsuitable for radical surgical treatment, have a worse prognosis than early-stage malignant PPLs, which are possibly treatable with surgical resection (lobectomy, segmentectomy, wedge resection) [26,36].

Patients frequently have an advanced stage of disease at the time of diagnosis due to the delay of symptom presentation, especially for PPLs, which impairs survival [37]. Fortunately, we are currently entering an era of lung cancer screening. The National Lung Screening Trial in the U.S. demonstrated the benefit on mortality with lung cancer screening derived from the use of low-dose high-resolution CT (HRCT) [38]. Subsequently, other studies have shown evidence to support this practice, which allows the detection of pulmonary neoplastic lesions in an early stage, very often represented by PPLs [8,9]. Thanks to these studies, it is currently possible to carry out an early diagnosis and therapy in lung cancer cases [39].

### 1.3. Initial Approach to PPLs

The first-line examination is either a bronchoscopy with tissue sampling or computed-tomography-guided transthoracic needle aspiration (CT-TTNA) [40]. Traditional bronchoscopic techniques have low diagnostic accuracy for most PPLs, especially the smallest ones. Notably, it has been estimated that the diagnostic yield of bronchoscopy for PPLs ≤2 cm is only 14% vs. 31% for those >2 cm [41]. CT-TTNA offers an alternative procedure for sampling PPLs with a reported diagnostic yield superior to 50% [42,43,44,45]. However, CT-TTNA is associated with a significant incidence of pneumothorax (45%), requiring chest tube placement in up to 15% of patients [46]. In addition, CT-TTNA does not allow mediastinal staging and has a non-negligible rate of false negative results (30%), mostly with small lesions [47,48,49,50]. For this reason, CT-TTNA alone may not rule out a malignant diagnosis [51].

Fluorine 18 fluorodeoxyglucose positron emission tomography/computed tomography (18F-FDG PET-CT) may add relevant information in the staging and risk stratification phase of a PPL, especially for solitary pulmonary nodules. It is considerably more likely that solid lung nodules larger than 8 mm in diameter that do not significantly uptake 18F-FDG are benign [52]. However, despite the usefulness of the examination, this is not a substitute for actual tissue sampling [53].

Notwithstanding, over the last years, several novel tools have improved the traditional endoscopic techniques for the diagnostic sampling and, eventually, staging of PPLs. Ultrathin bronchoscopy, radial-probe endobronchial ultrasound (RP-EBUS), virtual navigation bronchoscopy, electromagnetic navigation (EMN), shape-sensing navigation, augmented fluoroscopy, cone-beam computed tomography (CBCT), and robotic bronchoscopy (RB) are now game changers for the interventional pulmonologist (IP). As will be discussed in this review, these minimally invasive procedures aimed at both diagnosis and therapy of PPLs, have been recently introduced in real-life practice, and their safety and efficacy are still under evaluation. We strongly believe that these techniques will lead to a general improvement in the prognosis and personalization of the patient management path.

## 2. Methodology of the Review

This narrative review evaluates the existing literature by collecting the primary English language bibliographic references in international scientific databases (Medline, Pubmed, Scopus, and Google Scholar). The search strategy aimed to include the most significant documents dealing with the diagnosis of PPLs by using the following keywords in combination with the Boolean operator OR and: interventional pulmonology, bronchoscopy, lung nodule, peripheral lung lesion, lung cancer, lung cancer screening, endobronchial ultrasound, ultrathin bronchoscopy, lung navigation, robotic bronchoscopy, cone-beam computed tomography, and lung ablation. The interval considered by the research was from January 2009 to November 2022. Other references considered significant were included by the authors.

Systematic reviews with meta-analyses, randomized control trials (RCTs), and research were included in our review. We also reviewed the guidelines for diagnosing pulmonary lesions by the leading scientific societies.

To minimize bias, two authors (M.M., A.F.) independently carried out the search, deduplicated the research results, screened the articles, and selected the studies to be included in this review. Of note, the list of included references is not necessarily all-encompassing but reflects the body of evidence believed appropriate to the purpose of this document: highlighting the latest progress made in the endoscopic diagnosis and therapy for PPLs.

## 3. Endoscopic Identification of a PPL

When a PPL is identified, the IP decides whether and how to approach it based on the lesion’s morphology outlined on the chest CT scan [26,54]. CT patterns, such as the presence of a bronchus sign and a lesion size of >20 mm, are predictors of adequate sampling [55,56]. In addition, operator experience and high institutional procedural volumes positively impact the diagnostic yield and accuracy of PPLs [57].

During the navigation to the lesion and sampling procedure, the IP will consider all aspects aimed at maximizing the diagnostic yield: adequate sedation of the patient, collection of at least five bioptic samples, and the use of rapid on-site evaluation (ROSE) of the sample on slides set up in the endoscopic room. These considerations maximize the possibility of achieving success in reaching and withdrawing a significant sample from the lesion and minimizing the risk of repetition of the procedure due to insufficient tissue material [58,59,60,61,62].

The latest guidelines from the American College of Chest Physicians encourage using minimally invasive modalities [2]. Nonetheless, there is no clear evidence comparing the yield of these types of endoscopic techniques for diagnosing PPLs. Therefore, the choice between the techniques mainly depends on IP expertise and available technology.

All the technologies available for the IP may improve the performance of PPL sampling procedures (Table 1).

### 3.1. Ultrathin Bronchoscopy

In the past, flexible bronchoscopy (FB) did not allow direct visualization of the more peripheral airways, as the caliber of the instrumentation available for the adult patient was large. For this reason, since 1985, Prakash hypothesized using small-sized pediatric bronchoscopes to navigate to the periphery and to reach and sample PPLs [63]. However, conventional bronchoscopes tend to be large-sized (outer diameter >4 mm), making reaching the more distal bronchial branches difficult. For this reason, in recent years, ultrathin bronchoscopes have been developed.

Although a universal definition of ultrathin bronchoscopy does not exist, experts generally consider ultrathin bronchoscopes as those with an outer diameter of 2.8–3.5 mm [64,65]. The small size of ultrathin bronchoscopes allows passing through smaller airways to reach more peripherally located PPLs. In addition, dedicated miniaturized sampling instruments (e.g., needles and forceps) may be introduced through the small working channels to sample these lesions.

Even though the ultrathin bronchoscope can reach the most distal airways, it might not allow direct visualization of PPLs. For this reason, its use may be combined with RP-EBUS, fluoroscopy, and navigation systems to address this limitation. The diagnostic yield for the multimodal sampling of PPLs may exceed 70% and is strictly associated with the lesion’s size and the presence of the bronchus sign [66,67,68].

### 3.2. Endobronchial Ultrasound

Both convex-probe endobronchial ultrasound (CP-EBUS) and RP-EBUS may aid PPLs sampling. CP-EBUS allows direct observation and a contemporary minimally invasive sampling of anatomical structures beyond the airway walls [69]. It can be used in sampling PPLs in contact with the central airways: lesions in the upper lobes, close to the trachea, or the lower lobes, close to the main bronchi [70].

RP-EBUS represents a way to confirm the real-time positioning of the bronchoscope relative to the lesion to be sampled. By locating the area surrounding the PPL, RP-EBUS maximizes the sample quality and reduces the risk of iatrogenic pneumothorax [71,72,73]. Its use is often associated with other guiding technologies such as fluoroscopy [74,75], EMN [76], or CBCT [77]. A guide sheath may also be used to direct the path of the sampling tool between the bronchoscope and the lesion after retracting the RP-EBUS probe [78,79].

Overall, the diagnostic yield of bronchoscopy with RP-EBUS for PPLs has been estimated at around 69–73% [1,80]. As expected, the diagnostic yield strongly depends on the PPL’s size and the probe’s position relative to the PPL [80]. Nonetheless, several authors have consistently demonstrated that the yield for RP-EBUS guided sampling of PPLs is sensibly higher than that of traditional transbronchial biopsies [81].

According to the ACCP guidelines, RP-EBUS should be prioritized for the first sampling of PPLs [82].

### 3.3. Navigation Systems

FB aimed at sampling PPLs has been historically performed under fluoroscopic guidance [78]. However, new auxiliary navigation systems have progressively been developed to assist the IP in navigating within the bronchi. In fact, advancement and orientation become progressively more complex as the bronchoscope moves further into the periphery [83]. These systems rely on preprocedural CT images to obtain a three-dimensional (3D) airway model and a pathway. The former can be used for real-time navigation guidance within the airway to reach an estimated proximal zone to the PPL [84,85]. The significant advantages of these navigation systems are evident in cases where CT-guided biopsy or the classical fluoroscopy-guided bronchoscopy are not safe due to PPL-related factors (e.g., underlining emphysema, proximity to a major vessel) or in cases where previous sampling has been nondiagnostic, when a long procedure is needed for both lesion sampling and mediastinal staging, when sampling of multiple lesions is necessary, and to obtain large quantities of tissue for molecular analysis [86].

A significant limit of these technologies is the presence of a high discrepancy between the position of the PPL estimated by the navigation technology and its actual anatomical position. The difference between the PPL’s theoretical and actual position has been defined as CT-to-body divergence and represents a phenomenon explained by the dynamic condition of the lung parenchyma during the procedure [87]. The lung exhibits movements associated with ventilation and progressively, during the procedure, with zonal atelectasis [88].

#### 3.3.1. Virtual Bronchoscopy

The navigation technologies that have first been introduced in clinical practice are virtual bronchoscopy and electromagnetic navigation (EMN). Virtual bronchoscopy is a CT-based imaging technique that allows a noninvasive intraluminal evaluation of the tracheobronchial tree. In addition, the airways’ and carina’s mucosa morphology may be evaluated noninvasively, and airway exploration via imaging software may aid in the mental mapping of the route to the PPL [89].

Virtual navigation bronchoscopy (VNB; Archimedes System) uses the patient’s CT scan to represent the airway via pattern recognition software [90]. In other terms, VBN allows navigation to the PPL by simultaneously aligning actual bronchoscopy views and virtual views. Since VNB alone may not definitively confirm the localization of the target PPL, the virtual pathway derived from VNB may be coupled with fluoroscopy (fused fluoroscopic guidance) or RP-EBUS. [91] The primary benefit given by this technology is the 3D reconstruction of the vascular structures, theoretically allowing to reach a PPL by the transparenchymal route (bronchial transparenchymal nodule access), creating a path through the lung parenchyma while avoiding the vascular structures, even in the absence of a bronchus sing [92]. The accuracy of VNB systems strongly relies on the quality of the radiological data [93]. Overall, the diagnostic yield of VNB on PPL has been reported from 67 to 74% [93,94,95].

#### 3.3.2. Electromagnetic Navigation

EMN (superDimension, SPiN Perc System, Monarch) initial trials occurred in 2005 [96]. The technology is based on virtual bronchoscopy planning software and a “location board” emitting low-frequency electromagnetic waves. Results from the chest CT data are combined with the electromagnetic field board to guide the real-time tracking of endoscopic instruments to reach the PPL. Specifically, via an extended working channel, the locatable guiding probe is advanced in the airway with the help of a steerable catheter. The probe orientation is identified in 3D space through the electromagnetic field [97]. Likewise, in VNB, the procedure’s outcome depends mainly on the quality of the baseline CT investigation and the preprocedural mapping of the pathways leading to the target lesion. Current implementations include active correction of the EMN guidance system’s position with a standard C-arm fluoroscope (superDimension; Medtronic, digital tomosynthesis-electromagnetic navigational bronchoscopy) [98]. EMN may also be combined with RP-EBUS [99]. According to the two most extensive meta-analyses focusing on EMN, the overall diagnostic yield of EMN on PPLs ranges from 64 to 67% [100,101]. Sensible variability in diagnostic yields exists, depending on the lesion’s size, location, combination with RP-EBUS, radiological patterns, and sampling instruments [101]. Interestingly, the use of EMN has been proven safe even in the presence of pacemakers and defibrillators [102,103].

#### 3.3.3. Shape-Sensing Navigation

Shape-sensing navigation (ION, Intuitive Surgical) is a novel guidance system sold only in conjunction with the ION robotic bronchoscopy technology, which tracks the position of a dedicated articulating catheter [98]. A thin fiber embedded along the catheter’s entire length provides the basis for the system, measuring its length and shape hundreds of times per second while displaying its shape and position relative to the surrounding anatomy [104]. The guide allows strict closed-loop control to maintain the catheter in the correct position for sampling. The main advantage of this technology is the absence of magnetic fields and the absence of interaction with metal objects (e.g., pacemakers). To date, very few experiences with shape-sensing navigation have been reported. A premarketing study found that the ION system reached PPLs with a mean size of 14 mm in 96.6% of cases, with an overall diagnostic yield of 79.3% [105]. A small prospective analysis conducted in the U.S. demonstrated a diagnostic yield of 86% with shape-sensing navigation for PPLs with a mean diameter of <2 cm [106]. More recently, J. Reisenauer et al. found that the ION system allowed obtaining a sample of adequate quality in 95% of the samplings [107].

### 3.4. CBCT and Augmented Fluoroscopy

Implementing CBCT in clinical practice represented one of the last decade’s most critical advances in interventional pulmonology. This type of technology uses a rotating C-arm that generates intraprocedural images with a quality almost similar to a chest CT [108,109,110]. CBCT provides 3D reconstructions by combining coronal, sagittal, and axial views, which are invaluable during navigation. This system supports the physicians in confirming the intraprocedural location of the target PPLs [111]. In addition, the accurate real-time confirmation of the “tool-in-lesion sign” allows the confirmation of the instrumentation within the PPL and consequently guides tissue sampling [112]. Another essential feature of CBCT is the ability to detect atelectasis, which may limit the navigation and visualization of the target [111]. Eventually, CBCT can be combined with other techniques, such as ultrathin scopes, RP-EBUS, and EMN. It has been estimated that the diagnostic yields of CBCT combined with traditional bronchoscopy and EMN reach 70% and 87.5%, respectively [112,113].

Multiple companies have developed their version of both portable and fixed CBCT systems [111,114,115,116,117,118]. When considering using CBCT, physicians should remember that this technique causes significant radiation exposure, needs a complex workflow, and lengthens the procedural time [111]. For this reason, adequate training is required for its use, and a close relationship between the PI and the radiologist is indispensable. In managing the device, the specific procedural needs must be carefully balanced with the radiological exposure of the patient and the staff of the endoscopy room [119].

Augmented fluoroscopy (AF) is a novel technology that combines a baseline CT image derived from either a preprocedural chest CT or an intraprocedural scan performed by CBCT with real-time fluoroscopic guidance [120]. This technology is integrated into the processing software of some CBCT devices [121] or can be purchased separately (LungVision, Body Vision Ltd., Ramat Ha Sharon, Israel) [120,122]. This process provides 3D live fluoroscopic guidance. Through AF, the IP can localize the smallest PPLs dynamically and compensate for respiratory motion during navigation and sampling [121].

CBCT combined with AF has shown a significantly high diagnostic yield [123]. Recently, C.K. Lin et al. retrospectively reviewed the diagnostic yield of endoscopic procedures conducted with CBCT and AF (CBCT-AF) and compared them to those conducted without AF. As expected, the CBCT-AF group had a significantly higher diagnostic yield, especially for smaller lesions (68.8% in the CBCT-AF group vs. 0% in the non-AF group, *p* = 0.026 for lesions ≤ 10 mm, and 77.5% vs. 46.4%, *p* = 0.016, respectively, for lesions between 10 and 20 mm).

### 3.5. Robotic Bronchoscopy

Robotic endoscopic systems for bronchoscopy (RB) are experiencing a progressive global diffusion, limited, as for most iconic robotic technologies, by the high cost of acquiring the technology and its consumables [124,125].

Currently, only two platforms are available (Monarch and ION by Intuitive Surgical), with more to come [126]. Both devices’ steerable catheters have a small outer diameter, making it feasible for them to reach the peripheral areas of the lung, theoretically improving stability and control over traditional bronchoscopy and increasing diagnostic yield [126]. However, despite RB reaching deeper into the lung, lesion size still plays a role in diagnostic accuracy. The risk of complications is still high due to inexperience with the technique and the absence of widely shared international protocols on its use and the management of intraprocedural complications (e.g., bleeding) [127,128].

### 3.6. Others

Other promising technologies are awaiting final scientific support for their widespread implementation in clinical practice.

Confocal laser endomicroscopy (CLE) allows in vivo imaging of tissues. Alveolar spaces may be visualized with a miniprobe (Alveoflex), usually introduced in the lung periphery with a guide sheath [129]. This technique may be used before sampling a PPL to confirm the appropriate position of the probe relative to the lesion and allow in vivo cellular imaging by direct observation or after topical administration of specific dyes [129,130]. CLE-observed features might even identify lung carcinoma directly in ex vivo samples [131,132] and have been proposed as a substitute for ROSE [133].

Optical coherence tomography (OCT) is an imaging technique based on near-infrared light. The dedicated radial probe creates images using near-infrared light scattering with a 15 µm resolution. OCT provides an advantage over endobronchial ultrasonography because light waves can be used in air-filled anatomical compartments without requiring a transducing medium or direct contact with the tissue [134]. The system can directly generate two-dimensional images and carry out three-dimensional reconstructions using the dedicated software. The technology has only recently been introduced in the context of the assessment of PPLs [135]. Patterns visualized by OCT also correlate with PPL’s malignancy [136].

## 4. Treatment of PPLs with Endoscopic Ablation Techniques

Current guidelines recommend surgical resection for early-stage NSCLC in patients eligible for radical treatment [137]. However, the site of the disease and the patient’s comorbidities may make the patient unsuitable for surgery [138,139]. In fact, unresectable disease, typically represented by stage III and IV disease, may be approached surgically only in the context of salvage surgery or after a course of neoadjuvant therapy [140,141,142]. In addition, lung cancer patients, due to their age and exposure to risk factors such as cigarette smoking, are frequently affected by other diseases such as chronic obstructive pulmonary disease, heart failure, ischemic heart disease, arterial hypertension, atrial fibrillation, cerebrovascular disease, and peripheral vascular disease [143,144]. These pathologies may both exclude eligibility for surgery or worsen postoperative survival outcomes, a matter still neglected in the medical literature [145,146]. The only other option remaining with a radical intent is stereotaxic body radiation therapy, which, despite its effectiveness, bears significant adverse effects [147,148,149,150]. For all these reasons, endoscopic approaches have progressively become more investigated due to the reduced risk of short-term and long-term complications [151].

Potential uses for bronchoscopy include pleural dye marking of PPLs for surgical resection [152,153,154,155], placement of fiducial markers for radiotherapy [149,156,157], and direct ablation [158]. The latter is mainly an active research field, currently distant from clinical practice but rapidly progressing and supported by a large IP community.

Dye marking of a PPL has a practical utility in identifying lesions otherwise not visible or palpable by the surgeon, allowing shorter operating times and possibly carrying out minor resection operations, such as segmentectomies or wedge resections [155,159]. Indeed, the identification of small PPLs and confirmation of their complete resection can be challenging. Factors such as small size, ground-glass appearance on CT, and ample distance from the pleural surface make it difficult to identify a lesion intraoperatively [152,155]. The defect of this procedure lies in the fact that it must be carried out temporally close to the surgical procedure, often in the same operating room, as the dye may not have a long half-life [155]. The dyes currently available are methylene blue [155,160,161,162,163], indigo carmine [159], and indocyanine green [164,165].

Fiducial markers (FMs) for radiotherapy may be positioned with EMN or RP-EBUS guidance [149,156]. Generally, they are composed of a nitinol filament attached to a gold seed. Different types of FM are available: linear, coil-tailed, coil-spring, and two-band [149]. The main adverse event related to this procedure is the migration of the positioned marker [166,167].

Bronchoscopic ablation technologies (Table 2) include radiofrequency ablation (RFA), microwave ablation (MWA), photodynamic therapy (PDT), brachytherapy, cryoablation, laser ablation, bronchial thermal vapor thermal ablation, and direct therapeutic injection [158,168]. Many of these techniques are currently in the early stages of application in humans, and many clinical trials are being developed to study their efficacy in real life.

RFA uses an electromagnetic wave, delivering a thermal injury to the PPL via an electrode inserted directly into the lesion [169]. The therapeutic application of this technology is based on the assumption that cancer cells generally are more susceptible to heat than normal cells [169]. A high-frequency alternating current provokes coagulation of the tissue surrounding the electrode itself [170]. Due to the limited heatable radius, PPLs with a diameter smaller than 30 mm are best suited to this treatment. Given the current limitations of navigation technology for the periphery of the lung and the limited ability to place a catheter within a lesion located eccentrically to the afferent bronchus, RFA remains suitable for lesions with a bronchus sign on chest CT. New BTPNA access could offer a method to access lesions with no bronchus sign, and bronchoscopic RFA may be performed using this technology in the future [92]. Data on RFA of PPLs is still growing, but a recent study showed a local control rate higher than 80% and a 5-year survival rate of 60% after treatment [171].

MWA employs microwave frequency ranges between 300 MHz and 300 GHz to induce coagulative necrosis by heating the tissue at temperatures above 60 °C. The MWA antenna is usually set at a power level between 40 and 80 W, and the duration of exposure time ranges between 5 and 10 min for each PPL depending on its features (shape, size, and pattern). [172] Multiple human clinical trials are ongoing; see the respective National Clinical Trial (NCT) numbers: NCT05281237, NCT05532527, NCT04889989, NCT04369872, NCT04755738, and NCT05053802, with one having already delivered good preliminary results (NCT03603652) [173]. MWA produces a more extensive and uniform ablation zone than RFA because it generates a higher temperature in a shorter amount of time and is less susceptible to high impedance from high temperatures and heat sink effect [174,175]. Additionally, for MWA, direct ablation without saline infusion between the applicator tip and the target is possible for air-rich lesions such as ground-glass opacities (GGOs). As a result, MWA has benefits and excels at treating lung cancer that exhibits a GGO pattern [172,176].

PDT requires the preprocedural intravenous administration of a photosensitizing agent (a haematoporphyrin derivative) preferentially taken up by malignant cells [177]. Next follows the local endobronchial application of polarized light, usually red light at a 630 nm wavelength, which induces the intracellular production of reactive oxygen species, provoking local cell death [178,179]. Due to the difficulty of identifying a patient to whom it is appropriate to apply this technology and who can tolerate the adverse events related to the photosensitizing agent (e.g., sunburn), it remains scarcely widespread [177].

Brachytherapy involves the endoluminal administration of high-dose radiation therapy. This technology was previously applied to central airway malignancies but has been gradually abandoned. Only one study using this technology in the PPL research field has been completed (NCT00107172). Moreover, there are no currently ongoing clinical trials [180].

Cryoablation induces cell death using an alternation of freeze and thaw cycles. Although still not widely used, a Japanese trial has shown good survival following the cryoablation treatment of an inoperable NSCLC [181,182]. Treatment with cryoablation is particularly effective in poorly vascularized malignant lesions [183].

Laser ablation or bronchoscopic laser interstitial thermal therapy (BLITT) is an evolution of the classic treatment of centrally located endobronchial lesions [158,184]. A diode laser is applied by a laser delivery fiber (LDF) with a wide aperture, inducing tissue charring. The LDF may be advanced through the bronchial wall directly into the lung parenchyma. It was first evaluated in animal studies [184], and the first human trial ended recruiting this year (NCT03707925) with pending results.

Bronchial thermal vapor ablation entails the delivery of steam at a high temperature causing colliquative necrosis of the tissues distal to the treated area. Bacterial pneumonia and inflammatory pneumonitis following treatment are frequent [185]. It may be a potential treatment for localized lung cancer; however, the evidence is still unclear, and the first human trials are ongoing (NCT03198468 and NCT03514329).

Direct therapeutic injection aims at the intralesional administration of therapeutic drugs at high concentrations, limiting systemic administration side effects. The first evidence of the application of this technique dates back to 1980, using various chemotherapy agents. Current clinical trials aim to define whether direct therapeutic injection of immunotherapy or gene therapy mediated by a viral vector can improve quality of life and overall survival [186].

At present, most early human studies perform ablation followed by surgical resection. For individuals who are clinically inoperable, endoscopic ablation may be a therapeutic option with evident benefits over the treatments that are now available. Although many ablative methods have been put forth, most of the data has come from animal research. However, there are several human studies in progress globally. We can only hope that they will overcome the myriad difficulties that this innovative technology faces, so that it can eventually be used in the real-life setting [158].

## 5. Conclusions

The field of diagnostic and therapeutic bronchoscopy is ever-expanding. Whenever possible, instead of proceeding with peripheral navigation, it is preferable to resort to shorter and simpler procedures, such as sampling one or more mediastinal adenopathies through endobronchial ultrasound-guided transbronchial needle aspiration. However, when it is essential to reach for a PPL, the development of smaller endoscopic devices and improved navigation systems has led to the ability to reach deep into the lung. We strongly believe that, when used in conjunction, these tools may overcome the diagnostic yield plateau and reach better results. Currently, there is not a single modality or a standardized combination of modalities to be recommended.

The desirable goal is an integration of the techniques mentioned above based on the IP expertise, the characteristics of the lesions, and the consideration of patients’ comorbidities. Furthermore, the ultimate goal for every IP should be to provide an adequate diagnosis for a PPL with its staging within a single procedure and, in the future, a radical endoscopic therapy.

## Figures and Tables

**Table 1:** Comparison of navigation technologies for PPLs.

Method	Advantages	Disadvantages
Ultrathin bronchoscopy	Affordable Easily introduced into an already-established clinical practice	Operator-dependent tip stability Small working channel
RP-EBUS	Affordable Easily introduced into an already-established clinical practice	Need to interpret artifacts No real-time guidance during sampling
Virtual navigation bronchoscopy	Active navigation correction according to preprocedural planning BTPNA	CT-to-body divergence Absence of real-time position correction by fluoroscopy
EMN	Active navigation correction according to preprocedural planning Ability to associate real-time navigation correction with fluoroscopy Real-time guidance during sampling	CT-to-body divergence Interference with metallic objects and other magnetic fields
Shape-sensing navigation	Operator-independent tip stability	Expensive in terms of acquisition (at present only within robotic bronchoscopy technology)
Augmented fluoroscopy	Real-time navigation correction with fluoroscopy	Expensive in terms of acquisition
CBCT	The gold standard for tool-in-lesion confirmation	Expensive in terms of acquisition Non-negligible radiation exposure
Robotic bronchoscopy	Integration of a navigation system within an articulating catheter that can be used for multiple purposes	Expensive both in terms of acquisition and consumables Technical demanding Requires general anesthesia CT-to-body divergence

RP-EBUS, radial-probe endobronchial ultrasound; BTPNA, bronchial transparenchymal nodule access; EMN, electromagnetic navigation; CBCT, cone-beam computed tomography.

**Table 2:** Ablation technologies for PPLs and related mechanisms of action.

Method	Mechanism of Action
RFA	Electromagnetic-induced selective heat damage
MWA	Microwave-induced selective heat damage
PDT	Oxidative damage by a photosensitizing agent
Brachytherapy	Selective, high-dose radiation exposure
Cryoablation	Freezing-induced cytotoxicity and delayed cell apoptosis
BLITT	Heat-induced damage and charring
Thermal vapor ablation	Vapor-induced regional heat damage
Intralesional therapeutic drugs	Direct injection of an antineoplastic drug

RFA, radiofrequency ablation; MWA, microwave ablation; PDT, photodynamic therapy; BLITT, bronchoscopic laser interstitial thermal therapy; AEs, adverse events.

## Data Availability

Not applicable.
